# Revealing protein networks and gene-drug connectivity in cancer from direct information

**DOI:** 10.1038/s41598-017-04001-3

**Published:** 2017-06-16

**Authors:** Xian-Li Jiang, Emmanuel Martinez-Ledesma, Faruck Morcos

**Affiliations:** 1 Department of Biological Sciences, University of Texas at Dallas, Dallas, TX 75080 USA; 2 Department of Neuro-Oncology, The University of Texas MD Anderson Cancer Center, Houston, TX 77030 USA; 3 Center for Systems Biology, University of Texas at Dallas, Dallas, TX 75080 USA

## Abstract

The connection between genetic variation and drug response has long been explored to facilitate the optimization and personalization of cancer therapy. Crucial to the identification of drug response related genetic features is the ability to separate indirect correlations from direct correlations across abundant datasets with large number of variables. Here we analyzed proteomic and pharmacogenomic data in cancer tissues and cell lines using a global statistical model connecting protein pairs, genes and anti-cancer drugs. We estimated this model using direct coupling analysis (DCA), a powerful statistical inference method that has been successfully applied to protein sequence data to extract evolutionary signals that provide insights on protein structure, folding and interactions. We used Direct Information (DI) as a metric of connectivity between proteins as well as gene-drug pairs. We were able to infer important interactions observed in cancer-related pathways from proteomic data and predict potential connectivities in cancer networks. We also identified known and potential connections for anti-cancer drugs and gene mutations using DI in pharmacogenomic data. Our findings suggest that gene-drug connections predicted with direct couplings can be used as a reliable guide to cancer therapy and expand our understanding of the effects of gene alterations on drug efficacies.

## Introduction

Cancer, the second leading cause of death worldwide, is continuously affecting human health. To unravel cancer mechanisms and explore optimal therapeutic strategies, worldwide institutions have profiled various types of human tumors from patients and different tumor lineages. The cancer associated genetic, epigenetic, proteomic profiles enable researchers to explore and elucidate the molecular alterations and mechanisms underlying cancer progression. Several integrative analysis studies have been conducted on deciphering genetic and epigenetic data to understand the gene regulatory networks across tumor lineages^1, 2^. Additionally, the accessibility to proteomic profiles helps uncover important protein-protein interaction (PPI) pairs and the reconstitution of protein regulatory networks in cancer^3^. Understanding human diversity and disease-associated alterations at transcriptional and translational levels helps us uncover the events driving cancer processes, identify diagnosis biomarkers and therapeutic targets, and predict the prognosis of cancer patients.

Moreover, the concept of targeted therapy is proposed based on the distinctions in genetic landscapes between cancer cells and normal tissue cells. Hereditary and somatic mutations of cancer associated genes, such as *TP53*, *BRAF*, *ATM*, and *BRCA* 1 and 2, are commonly found in more than one type of cancers and are associated with poor clinical outcomes and chemotherapy resistance^4^. To overcome this challenge, targeted therapy drugs have been applied successfully and have achieved considerable breakthroughs. Erlotinib and Lapatinib, which selectively target *EGFR* mutants, have been extensively developed and approved to treat non-small cell lung cancer, whose clinical outcome is poor under traditional treatments, with higher survival benefit^5^. Imatinib, which targets the *BCR-ABL* translocation protein product, has tremendously improved the five-year survival rate to 89% in chronic myeloid leukemia (CML)^6^. Although treating cancers with targeted therapies by inhibiting or neutralizing cancer specific genetic alterations has been remarkable, there are still cases showing inert responses to these drugs. Recently, researchers believe that the efficacy of an anti-cancer agent in each patient is strongly affected or even determined by individual’s certain genetic features. Thus they have developed models to evaluate a patient’s response to certain drugs^7, 8^. For example, a *TP53* mutation impairs the efficacy of MEK inhibitors and MDM2 inhibitor^9^, while a *BRAF* mutation increases patients’ sensitivity to MEK inhibitors^10^. This idea supports the emergence of personalized medicine accompanied with pharmacogenomics^11, 12^. Studying pharmacogenomics benefits from a prudent prediction of drug response by using characterized genetic features, leading to controllable therapeutic strategies and predictable outcomes.

To address the increasing volume of cancer genomic, proteomic, and pharmacogenomic data, researchers have developed numerous approaches and statistical techniques, such as mutual information^13, 14^, regression^15^, Gaussian graphical models^16^, and entropy maximization^17^ among others. Mutual information (MI) is a local information theoretical metric used to compute the information dependence between two random variables. Many genomic analysis methods are derived from or include MI in their definition. MI based methods, such as ARACNE, CLR, and MRNET, are employed in gene and protein co-expression analysis and PPIs determination^3, 18^. However, mutual information is designed to uncover any kind of relationship among variables making it hard to distinguish between direct and indirect correlations among variables in a statistical model. To attack this problem, global statistical models have been proposed using approximate algorithms to alleviate computational complexity in signaling networks^19^. Direct-coupling analysis (DCA) has been developed as an efficient statistical inference method used for the study of co-evolution in protein sequences^20–23^. Direct Information (DI), a metric derived from DCA, is superior to mutual information in its ability to disentangle indirect correlations from direct correlations. An approximation of the global probability distribution of a large number of variables allows an accurate estimate of co-variations between two variables, such as pairwise residues within a protein chain, across multiple lineages or evolutionary history, while excluding secondary correlations between dependent variables^24^. DCA has been mostly applied in the field of structural biology^22, 25^ and system biology^26, 27^ through computing DI between pairwise residues based on the observed frequencies and inferred probability distribution of amino acids across the whole protein family. Here, we formulated a global model, which provides improved inference performance compared to MI based methods, aiming to reconstitute protein or drug-gene networks from noisy and large sets of genomic or proteomic data for the first time. We further propose that genetic signatures connected to patients’ response to drugs can be efficiently extracted from numerous biomarkers by employing a global method to infer drug-gene dependence in pharmacogenomic data, providing potential guidelines for personalized medicine.

In this study, to validate the performance of a model based on DCA on cancer profiling data, we used DI to predict protein interactions based on protein expression data from the Cancer Proteome Atlas (TCPA)^28^. A protein regulatory network is reconstructed based on the protein pairs with high DI values. We then evaluated the performance of our inference method on capturing predictive genetic features for drug response by extending the application of such methodology from proteomic data to pharmacogenomic data, obtained from the Cancer Cell Line Encyclopedia (CCLE)^8^, including gene mutation, drug response, as well as mRNA expression data. Analyzing the direct couplings between the mutation statuses of cancer associated genes in cancer cell lines and corresponding drug response gives us more predictive gene candidates for drug response.

## Results

**Figure 1:**
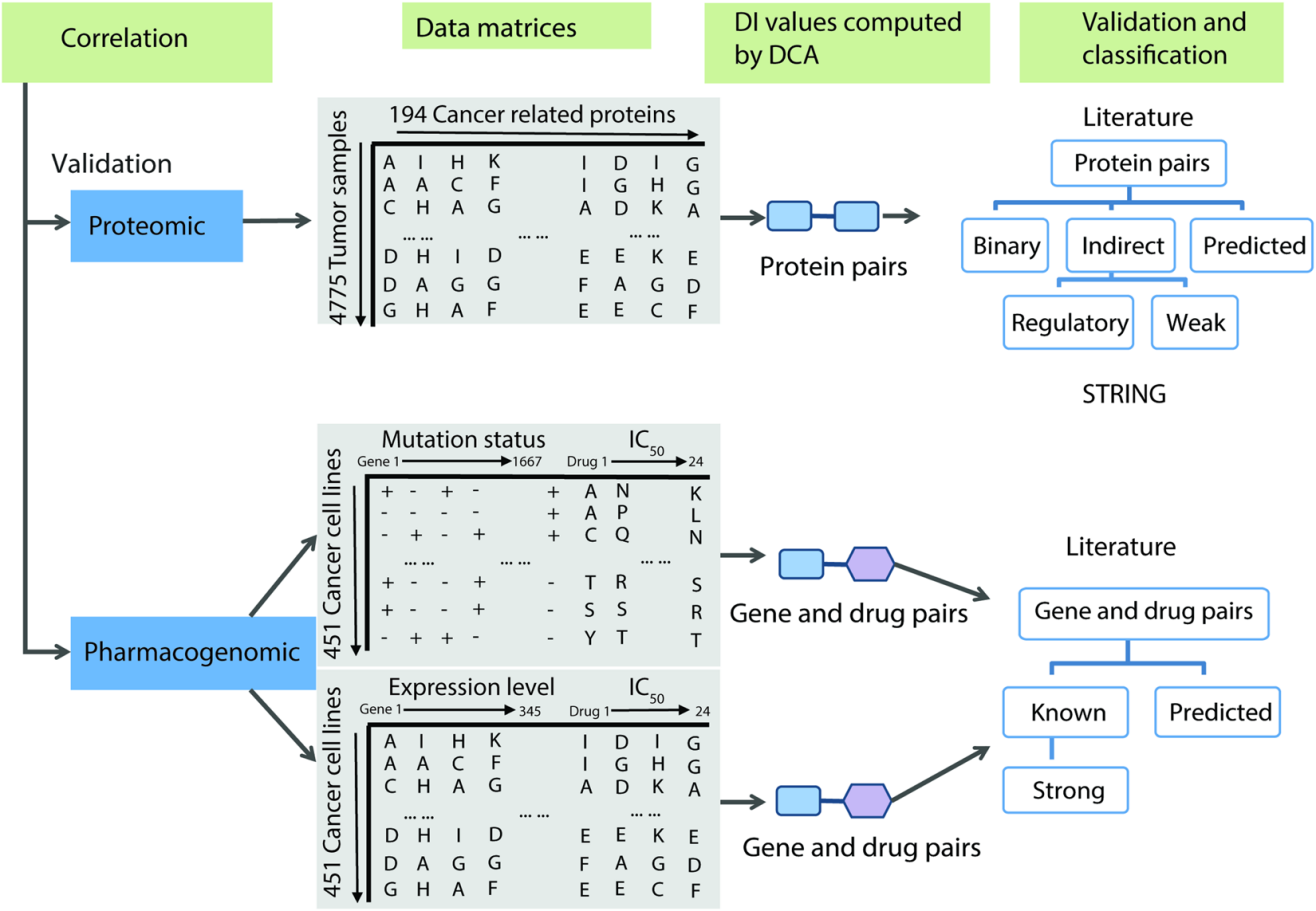
Workflow of direct coupling identification on cancer data. The proteomic data is obtained from the TCPA^28^, while pharmacogenomic data is acquired from CCLE^8^. The analysis on these data has been divided into 3 steps: (1) Expression and IC_50_ data is discretized to representative letters to form matrices with samples in rows and proteins, genes mutation statuses/expression levels, and IC_50_ values in columns, obtaining 3 types of matrices, the protein expression matrix, gene mutation and drug response matrix, and gene expression and drug response matrix. (2) Computation and subsequent descending ranking of DI values by using DCA and the matrices as input for each protein pair and gene-drug pairs. (3) Performance evaluation based on evidence searched from literature and classification of high confident pairs ranked top 100 based on the presence of evidence and strength of interaction.

The workflow of DCA on cancer proteomic and pharmacogenomic data involves three steps (Fig. 1): First, cancer data is processed and formatted into discrete matrices, a protein expression matrix, gene mutation-drug response matrix, and gene expression-drug response matrix. A detailed strategy for data processing is described in the Methods section. Then the DI values for all protein pairs and gene-drug pairs are computed by using those matrices as input for DCA and ranked, indicating a degree of direct statistical connectivity across tumor lineages. The higher ranked DI value indicates a stronger direct correlation. After that, the pairs with top ranked DI values are verified through literature research and then classified based on the type of interactions, indicating their strength and accuracy. Additionally, the verification of protein and protein interactions is also performed against STRING database^29^ to find additional evidence of known interactions. We analyzed the performance of our framework statistically and discussed the biological significance of resultant high confident prediction pairs. We built a pan-cancer protein coupling network and identified gene mutations strongly related with response of cancer cell lines to certain drugs.

### DCA recapitulates physical protein-protein interactions and protein coupling networks

DI values of 18,528 pairwise protein pairs are computed from this protein expression dataset using DCA and then ranked accordingly. The higher the rank of the DI value, the higher the confidence that such proteins are involved in physical or functional interaction. We obtained interaction evidence mainly from literature and using STRING as a secondary source to identify positive predictions among top ranked unique 100 protein pairs, which are approximately the top 0.5% of the total possible unique protein pairs. Literature evidence (PubMed IDs) and STRING scores for the 100 predicted pairs are listed in Supplementary Tables S2 and S3. We classified these pairs into different interaction types based on evidence from literature (Fig. 1). The PPIs in which two proteins are physically interacting with each other are classified as *binary interactions*. Other positive PPIs are classified as *indirect interactions* since they are not in physical contact but functionally related. The *indirect interactions* category is further divided into two types, *regulatory interactions* and *weak interactions*. The *regulatory interactions* category is limited to the pairs where one protein regulates the other protein’s level or activity directly, including the situation where two proteins are the main components of the same pathway. *Weak interactions* include PPIs where the two proteins are transcriptionally related, as well as other situations, *e.g*. two proteins share same regulatory intermediate protein. For protein pairs without support for its relationship, we defined them as *predicted interactions*. STRING interactions are annotated with aggregated scores and experimental scores, a value of 0.9 is the threshold for highest confidence, and 0.4 the threshold for medium confidence. We used combined scores with medium confidence as a support for true interactions and experimental scores with highest confidence as a support for physical interactions.

**Figure 2:**
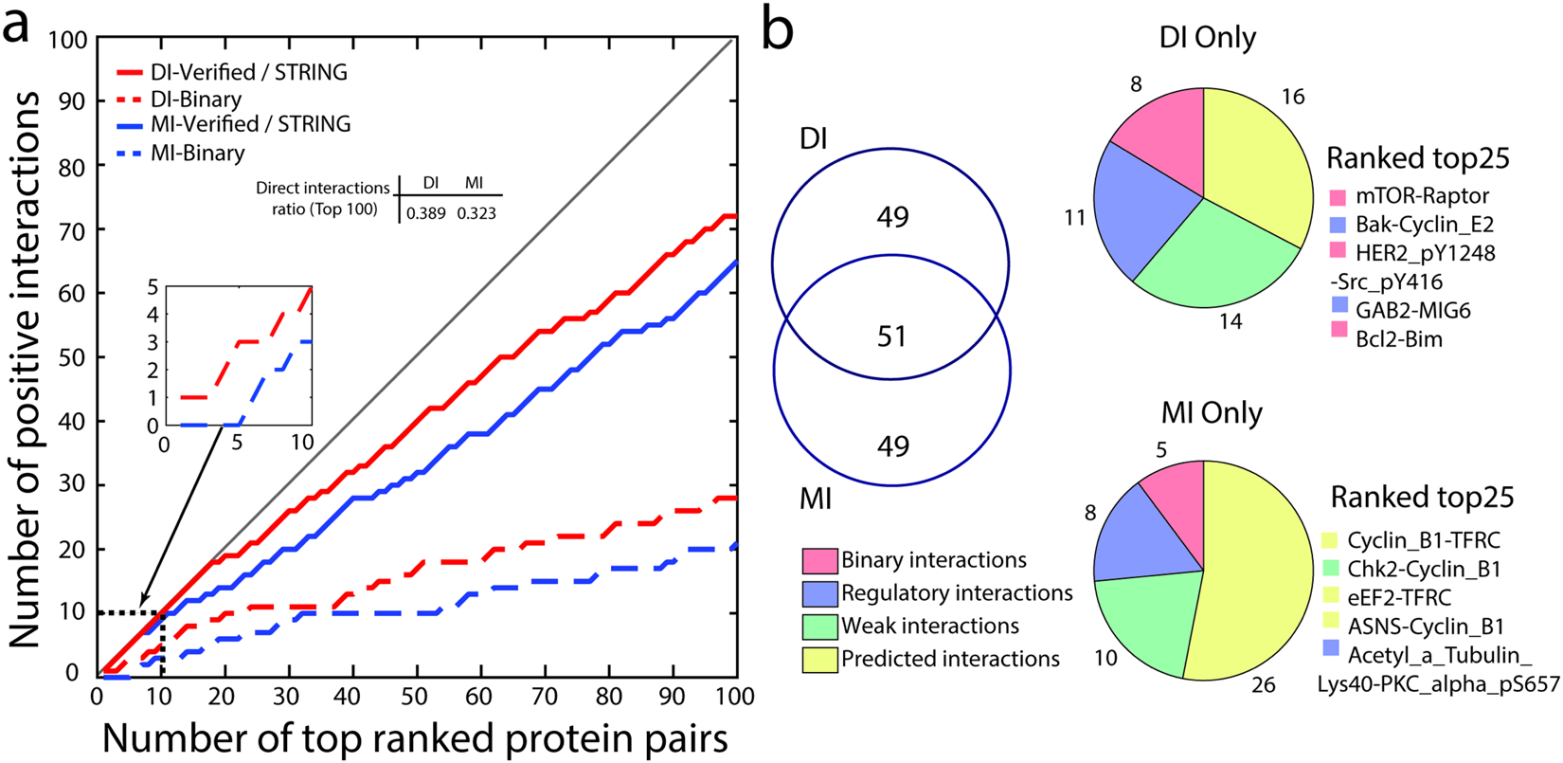
Protein-protein interaction pairs predicted from pan cancer protein expression levels with DCA. (**a**) Positive prediction values comparison between the direct information and mutual information. Solid line plots indicate number of protein-protein pairs verified with a curated literature review and STRING pairs with a combined scored at medium confidence (y-axis) against total number of pairs (x-axis) ranked by DI values (red) or MI (blue) values. Dash line plots indicate coupling pairs identified as *binary interactions* classified from a literature review and STRING pairs with experimental scores at highest confidence. The ratios of direct interactions to the all verified interactions for top 100 pairs are shown in the inset table. (**b**) Comparison of top 100 DI ranked and MI ranked predicted pairs. The pie charts on the right panel represent the distinct protein pairs in both groups with different colors indicating different categories. The digits near the pie charts are the number of protein pairs included into according category. Two lists named Ranked Top 25 show the detailed protein pairs that appear distinctly in the top 25 pairs by DI and MI methods.

Figure 2a shows that the positive prediction value (PPV) for DCA predictions on the protein expression data is as high as 72% for top 100 pairs, among which 28% PPIs are physically interacting, which are classified as *binary interactions* or experimentally scored with highest confidence in STRING. As a comparison, we also computed and ranked MI values for all possible protein pairs. The predictive performance of MI is lower compared to DCA, with PPV of 65%, including 21% physically interacting PPIs. The ratios of direct interactions to all positively predicted interactions for DI are higher than MI (Supplementary Fig. S2). When further looking into the top 10 pairs, the performance of DI on predicting physically interacting PPIs is as high as 50%, while MI only reached 30% of the accuracy. Moreover, MI is not able to identify physically interacting PPIs until the fifth pair. We observe that DI is better than MI in capturing interactions, especially in capturing pairs with highest confidence combined scores in the STRING database (Supplementary Fig. S3). Moreover, the area under precision-recall (P–R) curve (AUPR) for DI is higher than MI under both verification methods (Supplementary Fig. S4). The nominal p-value range for top 100 DI pairs is from 9.521E-35 to 1. 271E-13 (false discovery rate: FDR < 1.8E-11) (Supplementary Table S4, Supplementary Fig. S5). Figure 2b shows a comparison between the pairs uncovered by MI and DI and then verified in the literature. The DI group shares 51% of the top 100 pairs with MI set, resulting in 49 distinct PPIs respectively. A number of 33 pairs out of 49 pairs in DI set are predicted correctly with 8 *binary interactions*, while the PPV of MI is 23 out of 49. When comparing the distinct PPIs ranked among top 25 in each group, we find 5 non-overlapping pairs in each group. In the DI group, 3 pairs are physically coupled and 2 pairs are strongly related. However, 3 out of 5 pairs in the MI group are predicted, and none of the 5 PPIs are physically interacting.

**Figure 3:**
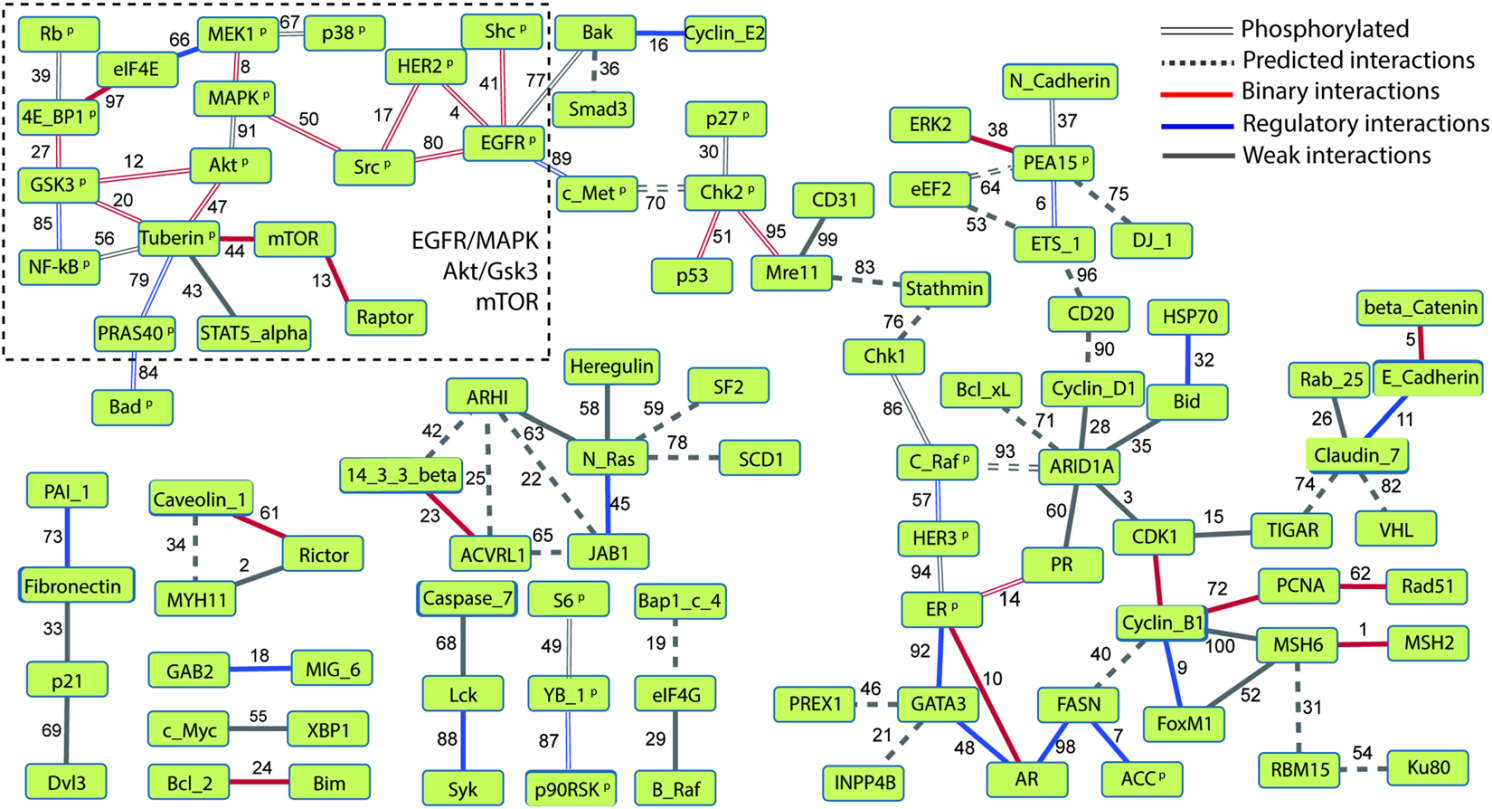
Visualization of pan cancer protein coupling network based on top 100 pairs ranked by DI values. Interactions are colored based on the interaction category, with red solid edges indicating *binary interactions*, blue solid edges indicating *regulatory interactions*, grey solid edges indicating *weak interactions*, and grey dashed edges indicating the *predicted interactions*. The double solid edges regardless of the color indicate that these interactions involve phosphorylated proteins with superscript of ‘P’. The edge coefficients represent the ranking by using direct information as a metric.

We explored the biological meaning of those highly ranked pairs and built a protein-coupling network (Fig. 3) by using the couplings with DI values ranked within top 100. There are 26 physically interacting protein pairs. As the protein-protein pair with highest DI value, MSH2 binds with MSH6 to form MutSα complexes and to modulate DNA mismatch repair^30^. Studies have shown that the heterodimer of EGFR and HER2 leads to a more activated EGFR state than EGFR homodimer, and thereby carries stronger tumorigenic effect^31^. The highest ranked EGFR and HER2 pair is the EGFR phosphorylated at Y1068 and HER2 phosphorylated at Y1248 (Supplementary Table S2). Indeed, 97% of cancers with HER2 phosphorylated at Y1248 exhibit detectable EGFR^32^ and HER2 stabilizes EGFR by reducing Y1068 phosphorylation^33^. Therefore, our methodology also exhibits a good performance on phosphorylated protein pairs and is able to distinguish the proteins with different phosphorylation sites.

The EGFR/MAPK, Akt/Gsk3/mTOR pathway crosstalk is also recovered in this protein-coupling network. As shown in the network, Src acts as an intermediate between tyrosine kinase receptors, EGFR and HER2, and MAPK pathway^34^. The network also contains the *regulatory interactions* between eIF4E and MEK1, known as the MEK/ERK/Mnk1/eIF4E signaling pathway^35^. The interactions between Akt and other proteins is also revealed in this crosstalk, since Akt activates mTOR-Raptor complex through Tuberous Sclerosis Complex 2, known as Tuberin^36^, and activates another downstream signaling molecule, GSK3. Additionally, NF-kB is found to relate with the Akt/Gsk3/mTOR pathway, which is supported by the fact that the activity of NF-kB is regulated by Gsk3^37^ and Tuberin^38^. Akbani *et al*. reported the mTOR pathway and Tuberin as two independent modules^28^, while in our work we find the strong connection between Tuberin and mTOR and classify this pair as a *binary interaction*. In one of the proposed models, Tuberin binds to its partner, Rheb, to inactivate mTOR^39^. Also a PC1/Tuberin/Rheb/mTOR complex model is proposed to illustrate the role of Tuberin in mTOR signaling pathway^40^. The network also exhibits the crosstalk between MAPK and Akt pathways^41^, which lacks the direct link found by Akbani *et al*. Moreover, the interplay among three hormone receptors, AR, PR, ER, is seen in our network, consistent with the presence of AR/ER/Src and ER/PR/PELP1 complexes^42, 43^.

In addition to the known PPIs, we found *predicted interaction* pairs, with plausible biological relevance. GATA3 and INPP4B are co-expressed in luminal breast cancer^44^. ARHI may connect to ACVRL1 through crosstalk between STAT3 and TGF β^45^, while ARHI binds to STAT3^46^ and ACVRL1 binds to TGF β^47^. These proteins may regulate the same biological process, indicating they are functionally or structurally related. ARHI has been shown to induce G1 cell cycle arrest in pancreatic cancer^48^, while JAB1 is physically contacting with CDKN1B^49^, a regulator of G1 progression. Bak and Smad3 both contribute to apoptosis regulation^50, 51^. The role of fatty acid synthase (FASN) blockage in cell cycle arrest indicates the potential interaction between FASN and cyclin-B1^52^. These protein pairs are worthy to be further investigated. We also validated the ability of highly ranked proteins used on the survival analysis for 15 different tumor lineages, from breast cancer to prostate cancer (Supplementary Fig. S6). We selected the top 20 proteins starting from the original highly ranked PPIs (Supplementary Table S4) for the analysis. Generally those 20 proteins are favorable at discerning clinical outcomes of patients with most types of tumors. The highest hazard ratio is 12.28 in LUSC. Furthermore, Supplementary Table S5 shows that DCA provides comparable or better survival models than using clinical covariates such as age and stage. Moreover, models combining proteins, age, and stage achieved better performance.

These results support the notion that proteins identified by our methodology are not only biologically relevant but also play a role in cancer mechanisms. We also found that DI captures true protein-protein pairs with high accuracy and exhibits a comparable capacity in studying biological correlations on the RPPA protein expression data. This study motivates a potential application of direct coupling analysis on continuous genomic and pharmacological profiling data.

### Gene mutation-drug response direct couplings reveal pathway/drug relationships

Given that DCA on cancer proteomic data provides meaningful biological connections, we then explored the use of global models on pharmacogenomic data. As one of the most important drivers for cancer, gene mutations play a key role in cancer initiation and progression. Mutations could not only provide the means for uncontrolled cancer growth but also can confer drug resistance. Recent datasets^7, 8^ contain both genomic and pharmacologic profiles that can be used to construct a statistical model connecting genes and drugs. We computed DI values for 40,008 gene-drug pairs and focused on top 100 ranked pairs, nominal p-values ranges from 2.326E-7 to 2.118E-5, (FDR < 5.9E-3) (Supplementary Table S11). This dataset incorporates mutation information of 1667 cancer associated genes (Fig. 1, Supplementary Table S6), with IC_50_ values for 24 drugs (Supplementary Table S7) across 451 cancer cell lines from 23 tissue types (Supplementary Fig. S7). Highly coupled variables represent gene-drug pairs with a drug response directly tied to the presence or absence of a mutation in a given gene. Therefore strong couplings will reflect a direct relationship between the gene and the effect of a given drug. Here, the relationships between genes and drug responses are divided into two major categories, *predicted interactions* and *known interactions*, based on the presence of experimental evidence for the relationship between the gene product and drug or the drug’s target(s). The *strong interactions* category is a subset of *known interactions*. The *strong interactions* category includes the gene-drug pairs where gene product is the direct target of the drug or affects the response to that drug, as well as the situations where gene or gene product is directly affected by that drug.

**Figure 4:**
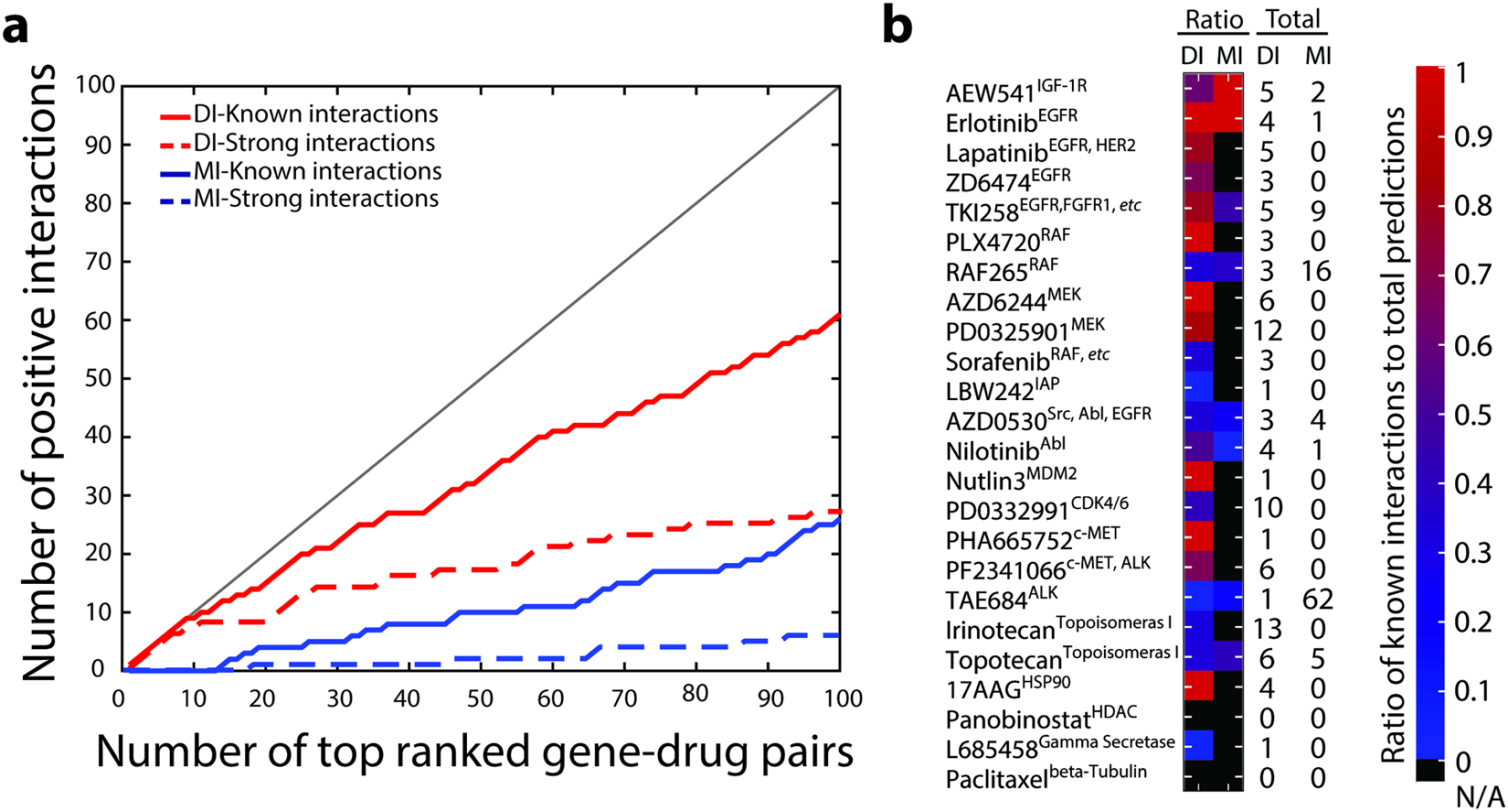
Direct Information as a metric in predicting gene mutation-drug response pairs. (**a**) Comparison between direct information and mutual information. Solid line plots indicate gene mutation-drug response pairs with *known interactions* (y-axis) against total number of drug-mutated gene pairs (x-axis) ranked by DI values (red) or MI (blue) values. Dash line plots indicate coupling pairs identified as *strong interactions*. (**b**) A heat map representation of ratios of *known interactions* to total predictions among pairs with top 100 DI values or MI values for each drug. The target(s) for each drug is shown in superscripts. The total numbers of gene mutation-drug response pairs captured by DI and MI for each drug are shown on the right of the heat map. Bars shown on the right in red denote high ratios and bars in blue depict low ratios. Bars in black indicate that the total prediction number in the top 100 pairs is zero.

As shown in Fig. 4a, for the top 10 DI ranked pairs, the PPV of relationships between mutated genes and drugs is as high as 90%, with 80% of the pairs classified as *strong interactions*. For the top 100 pairs the PPV is 61%, with PPV of 28% for *strong interactions*. A similar analysis is performed by evaluating correlations using MI. The MI predictions exhibit remarkably lower performance, with a PPV of 26% of *known interactions* and 6% of *strong interactions*. MI fails to capture *known interactions* in the top 14 pairs and *strong interactions* until reaching top 19 pairs. The precision-recall curve illustrates the marked difference in performance between DI and MI, with a difference of AUPR of 0.51 in favor of DI (Supplementary Fig. S8). These results suggest that DI is an effective metric at capturing the biological connections between mutated genes in cancer cell lines and their effects on drugs.

In addition to computing PPVs for of predicted gene-drug pairs, we also analyzed the PPV for individual drug among top 100 pairs (Fig. 4b). By comparing the diversity of drugs captured in the top 100 pairs ranked by DI values and MI values, we find that DI group exhibits a larger diversity of drugs uncovered in the top 100, while MI sheds more light on fewer drugs like TAE684, with 62 total pairs in the top 100 pairs, and RAF265, with 16 total pairs in the top 100 pairs. DCA uncovers more meaningful relationships on MEK inhibitors, AZD6244 and PD0325901, with ratios of *known interactions*/total pairs at 6/6 and 10/12 respectively. For the receptor tyrosine kinase inhibitors, AEW541, Lapatinib, TKI258, Erlotinib, and ZD6474, our framework obtains PPVs of 3/5, 4/5, 4/5, 4/4, and 3/5 respectively. Both RAF inhibitors are found 3 times in the top 100 pairs, however, the ratio of PLX4720 is as high as 1, while only one RAF265-mutated gene pair is regarded as *known interactions*. Our methodology captures only one pair of Nutlin 3 and of PHA 665752 with ratio of 1. When compared to the ratios predicted by MI for each drug, we observed worse performance of MI (Fig. 4b). Additionally, we compared our results for the drugs also uncovered by the elastic net regression (ENR) method used by Barretina *et al*.^8^, where all genetic features are used as input and activity area is used as a metric for drug sensitivity (Supplementary Fig. S9). We compared a maximum of 5 mutated genes connected to drugs by using the top 1% ranked links to achieve the largest drug coverage with respect to the ENR^8^. By calculating direct couplings we observe that for 4 drugs, AZD6244, Nutlin-3, PHA 665752, and 17AAG, we are able to infer correctly a 100% of their gene mutation-drug relationships while the ENR method does this for three drugs, PD 0325901, ZD6474, and PD 0332991. On the other hand, ENR finds mutation-drug relationships for 6 drugs where there is no evidence of connections for any gene, while with DCA only 1 drug has no supported pairs. These two methods find 5 overlapped gene and drug connections. The average positive prediction proportions for DCA is higher than ENR method with 64% and 41%, respectively. This result positively suggests that inferring direct couplings is a reliable alternative method for identifying gene mutations connected with drug response.

**Figure 5:**
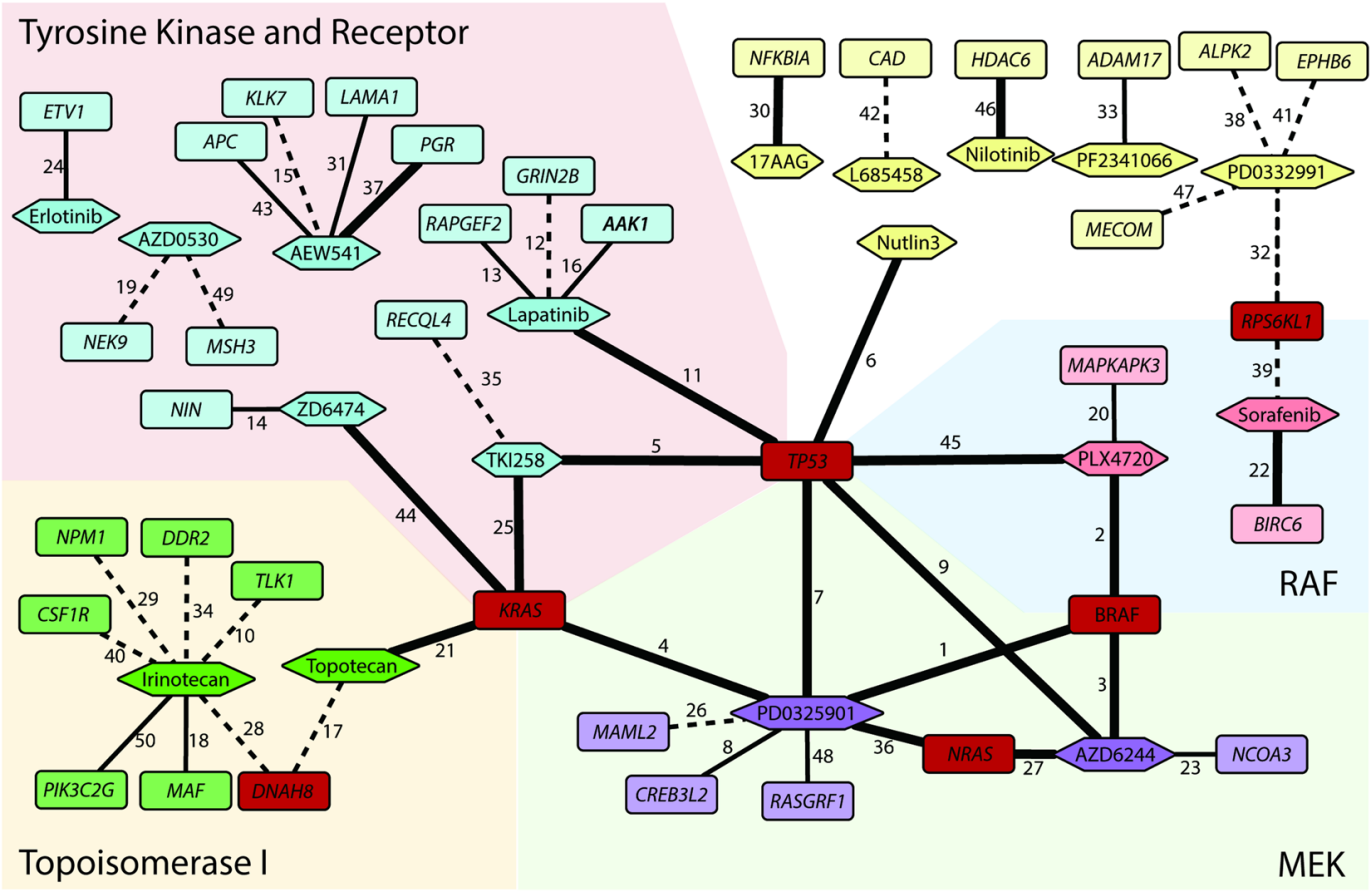
Top 50 drug-mutated gene relationships inferred with direct couplings. Clusters are annotated with different colors based on the pathways to which the targets of drugs belong. Rectangles represent drugs and ellipses are the mutated genes. Red nodes indicate hubs connected to several drugs. Solid lines indicate that the gene product is known to relate with the target of the drug it connects, defined as *known interactions*, with thick solid edges indicating *strong interactions* between drug and mutated gene. The thin solid lines indicate a weaker relationship between drug and mutated gene. Dashed edges show *predicted interactions* between drug and mutated gene that have not been found in the literature. The edge coefficients represent the rank using direct information as a metric.

To showcase the detailed gene mutations and drug response relationships uncovered by our methodology, we depicted the top 50 gene mutation-drug pairs in the Fig. 5, with 18 drugs involved. Based on their specific target(s), the drugs are classified as tyrosine kinase and receptors, RAF, MEK, Topoisomerase I, and one last group, which is not limited to only one specific type of target(s). Using direct information we capture a total of 19 gene mutation-drug response pairs with *strong interactions*. Among these 19 pairs, we found one mutated gene whose encoded product is the direct target for the drug, the *BRAF* and PLX4720 pair. Meanwhile, *BRAF* is predicted to highly relate with the other two MEK inhibitors, PD0325901 and AZD6244, with DI values ranking at position 1 and 3 respectively. There is evidence of a *BRAF* mutation being involved in the response to the MEK inhibitors since RAF triggers the MEK signaling pathway^53^. Also, a *BRAF* mutation has been used to predict the sensitivity to MEK inhibitors^10^. As the activators of RAF, *KRAS* and *NRAS* mutations are also highly connected with MEK inhibitors, AZD6244 and PD0325901. Indeed, the ability of using *KRAS* and *NRAS* mutations to predict the response to MEK inhibitors has been reported by other groups^54, 55^. We observe that *KRAS* is also related with two RTKs inhibitors, ZD6474 (the EGFR inhibitor) and TKI258 (inhibitor for FGFR, VEGFR, and PDGFR, etc.). Ligand mediated RTK activation transduces the extracellular signal and triggers intracellular cascade kinase signaling pathways, including the RAS/RAF/MEK pathway, leading to uncontrolled cancer cell growth. We have identified these two pairs as s*trong interactions*. Interestingly, a mutation of *KRAS* indeed affects the response to EGFR inhibitor^56^, while the efficacy of TKI258 in colorectal cancer is not dependent on the mutation status of *KRAS* we do find a connection in the DCA results^57^, indicating a possibly distinct effect of *KRAS* mutation status on TKI258 efficacy in other types of cancer. Additionally, a *KRAS* mutation status also affects the sensitivity of cancer cells to topoisomerase I inhibitor^58^ and its relation with Topotecan is captured by our framework and is classified as a *strong interactions*.

Notably, Fig. 5 shows *KRAS*, *BRAF*, and *NRAS* as genetic hubs. The most connected hub is, *TP53*, showcasing the well-known important role of *TP53* in cancer. *TP53* mutation has frequently occurred in most types of tumors and contributes to the initiation and progression of cancer^59^. The presence or absence of mutations relate with the resistance or sensitivity to many drugs, such as the MDM2 inhibitor, RAF/MEK inhibitors, and RTK inhibitors^9^. We find that *TP53* mutation is associated with 6 anti-cancer drugs in our analysis, including 2 MEK inhibitors, 2 RTK inhibitors, Nutlin3, and an RAF inhibitor, substantiating that *TP53* plays a key role in the determinant of drug sensitivity. For example, p53 protein forms a complex with the target of Nutlin-3, MDM2, to achieve the MDM2-p53 interaction loop^60^. In the other 4 gene mutation-drug response pairs characterized with *strong interactions*, *BIRC6* knockout affects the sensitivity to Sorafenib^61^, the activity of NF kappa B pathway is altered by the administration of 17AAG^62^, HDAC6 inhibition promotes the degradation of Bcr-Abl, the target of Nilotinib^63^, and PGR forms a complex with the AEW541 target, IGF1R^64^.

For the gene mutation-drug response pairs with *weak interactions*, most genes relate to drugs via regulatory roles. For example, *CREB3L2* ^65^, *NCOA3* ^66^, *ETV1* ^67^, and *MAPKAPK3* ^68^ are involved in and regulated by MEK signaling pathway. Also, some gene products are involved with certain pathways where the drug targets are in by some intermediates, such as NIN and ZD6474 through GSK3β^69^. Also, DCA uncovers 17 predicted gene mutation-drug pairs without support from experimental data. AZD0530 is a tyrosine kinase inhibitor, while NEK9 protein binds to NEK7 and releases its auto-inhibitory tyrosine kinase motif^70^. Our data suggests a potential unknown target for AZD0530.

**Figure 6:**
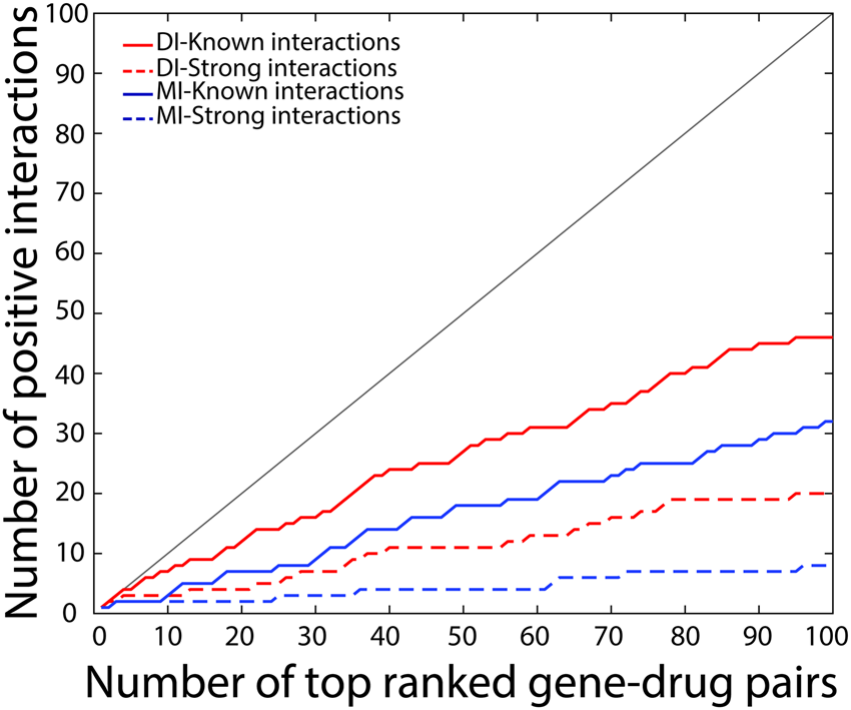
Prediction of gene expression and drug response pairs using DI and MI. Solid line plots indicate gene expression-drug response pairs with *known interactions* (y-axis) against total number of gene-drug pairs (x-axis) ranked by DI values (red) or MI (blue) values. Dash line plots indicate coupling pairs identified as *strong interactions*. DI yields better performance results at predicting gene-drug relationships compared to MI.

We also identified potential genes from expression data that are highly coupled to drug responses by computing DI values for 8,280 pairs. The performance difference between DI and MI for gene expression and drug pairs is similar as for protein pairs (Supplementary Fig. S10), with nominal p-values for top 100 pairs ranging from 1.937E-19 to 2.963E-10 (FDR < 2.5E-8). We recognized 46 gene-drug pairs out of total top 100 pairs (Supplementary Table S10) as *known interactions* supported by experimental evidence, among which 20 pairs are identified as *strong interactions* (Fig. 6). We analyzed the biological meaning of top 50 gene expression-drug pairs, which involve 18 drugs (Supplementary Fig. S11). The expression of 9 genes is strongly correlated with certain drugs. The protein MDM2 is the direct target of Nutlin-3, and this relationship is characterized with the highest DI value.

Computing direct couplings exhibits accurate performance in capturing drug response relevant genes. We not only identify clinically important gene markers for cellular response to multiple drugs, but also provide novel potential correlated gene mutations for those drugs with high ranking of DI values. In addition, the co-variations between some predicted or weakly interacted gene mutations and drug response imply that those genes may play key functional roles with the drugs or the pathways where the drug target(s) are involved.

## Discussion

In this study, we have demonstrated that by modeling protein and gene expression profiles as well as gene mutation and drug sensitivity data using a global probability distribution inferred by DCA, it is possible to reconstitute important molecular relationships related to cellular and cancer biology. The usefulness of DI as a metric of coupling is more evident when studying gene mutations and drug sensitivity, this is especially important since these datasets have been explored less than protein and gene expression data. Here we provide evidence that DI is a more appropriate tool to study the influence of mutations to drug sensitivity. Our methodology can also be applied to an ever increasing number of samples to generate more confident predictions and more diverse cancer associated data, such as copy number variation, epigenetic modification, miRNA expression, to name a few, in order to extract directly connected genetic features for anti-cancer drug efficacy. Given that the gene space is extremely large, our model included a subset (345) of cancer genes with expression levels in the study instead of the complete set of 18,988 genes. In the future, this computational challenge can be tackled by improving the efficiency of the matrix calculation and storage. This could allow us to build models with many more parameters and include more experimental data, such as the mRNA levels for whole genomes. With more tumor tissues or cancer cell samples profiled, tissue specific analysis is feasible. We expect to combine this systems approach with a molecular approach to study protein interfaces in drug design^71, 72^ in order to find possible compounds connected to target(s).

We have identified several well-known mutations that affect anti-cancer drug response, such as those in *TP53*, *BRAF*, *NRAS*, *KRAS*. In this analysis, the cellular response to Nutlin3, is found to be related with *MDM2* expression and *TP53* mutation. This observation is consistent with the knowledge that MDM2 is the direct target of Nutlin3 and MDM2 physically interacts with p53 to regulate p53, and also consistent with other published results^8^. We also identify genes not previously identified nor predicted to relate with drug response while they functionally interact with drug target, such as *HDAC6*-Nilotinib, *PGR*-AEW541. Our methodology will be able to find more relevant genes connected to drug response as more biological and pharmacological data become available. It’s also worthwhile to further study the gene-drug pairs classified as *predicted interactions*. In some instances, these connections could explain some heterogeneity in clinical outcomes across patients and variations in cellular processes by linking some novel proteins or gene-drug. This study explores how proteomics and genomics data can be analyzed in a distinct way to link high throughput profile data to clinical and pharmacological facts, including the prognosis prediction by selected factors and the genomic abnormalities related to the sensitivity of human cancer cell lines to diverse cancer drugs.

## Methods

### Cancer proteomic and pharmacogenomic data

The pan-cancer reverse phase protein array (RPPA) dataset, referred as PanCan16 was retrieved from The Cancer Proteome Atlas (TCPA) database^28^. This dataset contains protein expression data for 190 proteins, among which 52 are modified by phosphorylation and 1 is modified by acetylation, across 4776 tumor samples. A total of 16 tumor types are involved in this dataset, including breast invasive carcinoma (BRCA), lung adenocarcinoma (LUAD), lung squamous cell carcinoma (LUSC), bladder urothelial carcinoma (BLCA), colon adenocarcinoma (COAD), glioblastoma multiforme (GBM), head and neck squamous cell carcinoma (HNSC), ovarian serous cystadenocarcinoma (OVCA), rectum adenocarcinoma (READ), kidney renal clear cell carcinoma (KIRC), and uterine corpus endometrioid carcinoma (UCEC), Brain Lower Grade Glioma (LGG), Thyroid carcinoma (THCA), Stomach adenocarcinoma (STAD), Skin Cutaneous Melanoma (SKCM), Prostate adenocarcinoma (PRAD). The dataset has been processed and normalized with replicate-based normalization since the PanCan16 patient samples were profiled in different batches. The pharmacogenomic data, including mRNA expression, hybrid capture sequencing mutation, and a pharmacological profiling drug data were obtained from the Cancer Cell Line Encyclopedia (CCLE) database^8^. A number of 451 cancer cell lines are profiled for mRNA levels of 18,988 genes within which we selected 345 cancer associated genes, mutation status of 1667 cancer genes, as well as the pharmacological responses to 24 anti-cancer drugs, among which we used IC_50_ values in the study.

### Data Processing

Since DCA models the coupling between large sets of discrete variables we processed continuous data and transformed it into discrete representations for expression, sensitivity data (Fig. 1). The discretization strategy was designed to balance resolution and complexity. A uniform quantization seems to work better than an adaptive discretization but the method performance is robust to different discretization criteria. For the gene mutation dataset, the mutated status is denoted as ‘+’ and non-mutated status is denoted as ‘−’, in a matrix where rows are cell lines and columns are genes. This mutation matrix is then concatenated with IC_50_ data matrix after the IC_50_ values are quantized into 20 discrete states evenly, obtaining a 451 × 1,691 matrix with 451 cancer cell lines in rows and 1,691 columns of mutation status from 1,667 genes and IC_50_ values for 24 drugs. For the gene expression dataset, the continuous expression levels are mapped to 13 discrete states with unitary increments, followed by the concatenation with the discrete IC_50_ data matrix. As a result, the gene expression-drug matrix contains 451 rows of cancer cell lines and 369 columns of 345 profiled genes and 24 IC_50_ values. For the protein expression data, since the range for the expression level is 9, we divided the protein expression levels to 9 discrete states with unitary increments. Different quantization schemes yield similar performance results. The protein input matrix for DCA computation consists of 4,776 rows of patient samples and 190 columns of proteins.

### Null model construction and statistical tests

Three null models were built for protein expression, gene mutation-drug response, and gene expression-drug response datasets, respectively. To build these null models, the original dataset matrices (protein input matrix, gene mutation-drug matrix, gene expression-drug matrix) were shuffled by randomly switching columns and rows for each data point, and then fitted to a probability distribution. The nominal p-values for DI scores were computed by a left tailed z-test since the higher DI values (lower –ln(DI)) indicate higher confidence (see Supplementary Fig. S5) and then corrected using a false discovery rate (FDR) method as proposed by Benjamini and Hochberg^73^.

### Direct Coupling Analysis (DCA)

DCA infers in an efficient manner the parameters of a large join probability distribution and uses these inferred parameters to determine estimates of the coupling between pairs of variables in such distribution^23^. In the context of our work, DCA computes the amount of direct information (DI) between the columns in the matrices of proteins and gene mutations/expressions and drug response. In these matrices, the rows represent different cell lines or cancer tissues. As a reference, we also computed a standard measure of statistical coupling called mutual information (MI), which is computed by using reweighted frequency counts^23^:

1 $$M{I}_{ij}=\sum _{{x}_{i}{x}_{j}}{f}_{ij}({x}_{i},{x}_{j})\,\mathrm{ln}\,\frac{{f}_{ij}({x}_{i},{x}_{j})}{{f}_{i}({x}_{i}){f}_{j}({x}_{j})}$$

In equation (1), *i* and *j* denote columns in the input data matrix, while *x*_*i*_ and *x*_*j*_ denote the discrete variable values at column *i* and *j*. DCA is able to disentangle direct and indirect couplings by inferring a statistical model $P({x}_{1},\mathrm{......},{x}_{n})$ with the constraints that empirical frequency counts are the marginal probabilities,

2 $$\forall i,{x}_{i}:\sum _{\{{x}_{k}|k\ne i\}}P({x}_{1,}\mathrm{......},{x}_{n})\equiv {f}_{i}({x}_{i})$$

3 $${\rm{\forall }}i,j,{x}_{i},{x}_{j}:\sum _{\{{x}_{k}|k\ne i,j\}}P({x}_{1,}......,{x}_{n})\equiv {f}_{ij}({x}_{i},{x}_{j})$$

Another assumption is that the distribution of natural proteomic and pharmacogenomic data is estimated as a Boltzmann distribution, which is the most general and least-constrained model derived from maximum entropy modeling:

4 $$P({x}_{1},\mathrm{......},{x}_{n})=\frac{1}{Z}\exp \{\sum _{i < j}{e}_{ij}({x}_{i},{x}_{j})+\sum _{i}{h}_{i}({x}_{i})\}$$

In this equation, Z is the partition factor, defined as

5 $$Z=\sum _{{x}_{1},\mathrm{......},{x}_{n}}\exp \{\sum _{i < j}{e}_{ij}({x}_{i},{x}_{j})+\sum _{i}{h}_{i}({x}_{i})\}$$

and ${e}_{ij}({x}_{i},{x}_{j})$ is a matrix containing the direct coupling of two columns, while *h*_*i*_(*x*_*i*_) represent local biases of single variables. The pairwise couplings, ${e}_{ij}({x}_{i},{x}_{j})$, are estimated as:

6 $${e}_{ij}({x}_{i},{x}_{j})=-{({C}^{-1})}_{ij}({x}_{i},{x}_{j}),$$

where

7 $${C}_{ij}({x}_{i},{x}_{j})={f}_{ij}({x}_{i},{x}_{j})-{f}_{i}({x}_{i}){f}_{j}({x}_{j})$$

A complete derivation of this result can be found in ref. 23. Then the direct information (DI) between two variables can be estimated based on the direct coupling parameters. Similar as with MI, DI represents a metric between two columns, *i* and *j*, that is a result from direct correlation:

8 $$D{I}_{ij}=\sum _{{x}_{i},{x}_{j}}{P}_{ij}({x}_{i},{x}_{j})\,\mathrm{log}\,\frac{{P}_{ij}({x}_{i},{x}_{j})}{{f}_{i}({x}_{i}){f}_{j}({x}_{j})}$$

*i* and *j* are the positions of the proteins, with 0 < *i* < 191 and *i* < *j* < 191, for prote*i*n expression data matrix. For the case of gene expression and drug responses, *i* represents gene and *j* represents drug, with 0 < *i* < 346 and 345 < *j* < 370, in the gene-drug response matrix. For the mutational dataset, *i* represents the position of the mutated gene and *j* the index of the drug for which we have response data. The range for *i* and *j* is 0 < *i* < 1668 and 1667 < *j* < 1692, for gene expression-drug response data matr*i*x.

In equation (3), ${f}_{i}({x}_{i})$ and ${f}_{j}({x}_{j})$ are the empirical frequency counts, while ${P}_{ij}({x}_{i},{x}_{j})$ is the two-site model isolated from the above Boltzmann distribution model after introducing the direct coupling parameter, ${e}_{ij}({x}_{i},{x}_{j})$:

9 $$P({x}_{i},{x}_{j})=\frac{1}{{Z}_{ij}}\exp \{{e}_{ij}({x}_{i},{x}_{j})+{h}_{i}({x}_{i})+{h}_{j}({x}_{j})\}$$

The MI and DI values for the protein pairs or the gene-drug pairs are ranked with higher values indicating stronger connections, with zero value indicating statistical independence. For more details about the formulation and derivation of DCA please refer to Morcos *et al*.^23^.

### Performance evaluation

After predicting the highly coupled protein pairs or gene-drug pairs, we evaluated the performance of the prediction by searching for interaction evidence in the literature. To determine if a prediction is correct or not, we took experimental evidence as a benchmark. If two proteins interact physically with each other and experimental data is available, we designated it as a *binary interaction*. If one protein regulates another protein’s level or activity directly, two proteins share a same binding partner, or these two proteins are key factors in the same pathway even though they are not adjacent, we classified them as *regulatory interactions*. The remaining predictions, where two proteins relate to each other through transcriptionally regulation or involve remotely in the same or crosstalk pathways, are identified as *weak interactions*. *Regulatory interactions* and *weak interactions* together are classified as *indirect interactions*. For those protein pairs without experimental evidence, we defined them as *predicted interactions*. We also used STRING as a second source for interaction verification by using combined scores and experimental scores with medium (≥0.4) and highest confidence (≥0.9).

For gene-drug pair evaluation, the strategy is different. The *known interactions* are pairs with evidence from experimental data indicating that the gene product relates to the drug or the drug-involved pathway. Among the *known interactions*, the *strong interactions* represent the situations where a gene product is the direct target of the drug, the gene is known to affect the response to that drug, or the gene or gene product is interfered by that drug according to the literature. The remaining *known interactions* are recognized as weaker interactions. For those pairs without experimental evidence, we define them as *predicted interactions*.

### Precision-recall curves

The P–R curve was generated by cumulatively increasing the total prediction numbers, *i.e.* the ranking of pairs, from 1 to 100 to be the threshold for cutoff of predictions in the 100 pairs dataset (since we mainly focus on the top 100 pairs for all the three datasets). At each iteration, precision and recall values were computed. Precision represents the number of correctly predicted interactions (validated by literature evidence or STRING database) to the number of all predicted interactions, which is the iteratively increasing ranking number. Recall is defined as the number of correctly predicted interactions to the number of all interactions annotated as true by literature or STRING database, which is a fixed number of true/known interactions among top 100 pairs.

### Survival analysis

Analysis of survival was performed using the R statistical computing platform^74^. We used protein expression to fit a Cox model. Low and high-risk groups were obtained splitting the prognostic index, linear predictor from the Cox model^75^, by the median. The log-rank test was used to assess statistical significance of the difference between low and high-risk groups^76^.

### Data availability statement

Relevant input and output datasets used in the preparation of this study are publicly accessible in the following permanent link: http://utdallas.edu/~faruckm/PublicationDatasets.html.

## Electronic supplementary material

Supplementary Figures S1-S11

Datasets 1-11
