# Supplementary material for: Revealing protein networks and gene-drug connectivity in cancer from direct information
Source: Sci Rep. 2017 Jun 16;7:3739. doi: 10.1038/s41598-017-04001-3 (PMC5473890; doi:10.1038/s41598-017-04001-3)
Supplement: Supplementary file 1 — Supplementary Figures S1-S11 [file 41598_2017_4001_MOESM1_ESM.pdf]

Revealing protein networks and gene-drug connectivity in cancer from direct information

Xian-Li Jiang[1], Emmanuel Martinez-Ledesma[2], Faruck Morcos[1, 3]*

[1]Department of Biological Sciences, University of Texas at Dallas, Dallas, TX75080, USA.

[2]Department of Neuro-Oncology, The University of Texas MD Anderson Cancer Center, Houston, TX77030, USA.

[3]Center for Systems Biology, University of Texas at Dallas, Dallas, TX75080, USA.

*To whom correspondence should be addressed: Faruck Morcos (faruckm@utdallas.edu)

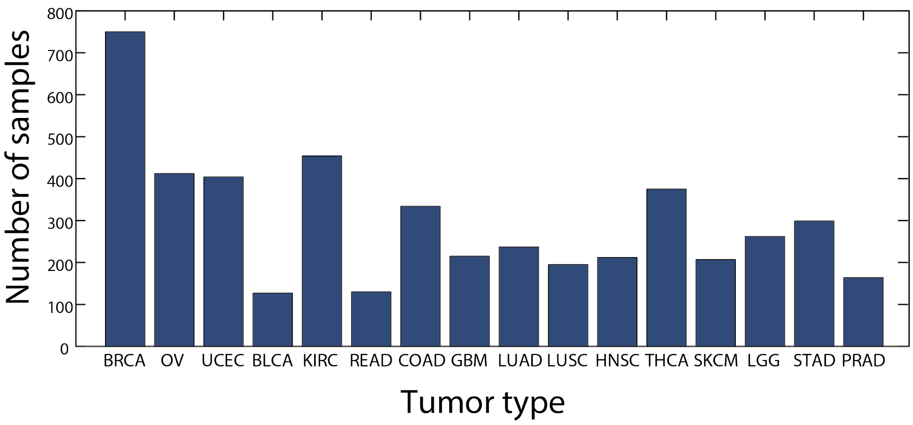

Figure S1. Sample number of 16 tumor types in TCPA dataset. DCA analysis is conducted on protein expression levels from 16 types of tumors (x-axis) with certain number of samples (y-axis). The 16 tumor types include: breast invasive carcinoma (BRCA), lung adenocarcinoma (LUAD), lung squamous cell carcinoma (LUSC), bladder urothelial carcinoma (BLCA), colon adenocarcinoma (COAD), glioblastoma multiforme (GBM), head and neck squamous cell carcinoma (HNSC), ovarian serous cystadenocarcinoma (OVCA), rectum adenocarcinoma (READ), kidney renal clear cell carcinoma (KIRC), and uterine corpus endometrioid carcinoma (UCEC), brain lower grade glioma (LGG), thyroid carcinoma (THCA), stomach adenocarcinoma (STAD), skin cutaneous melanoma (SKCM), prostate adenocarcinoma (PRAD).

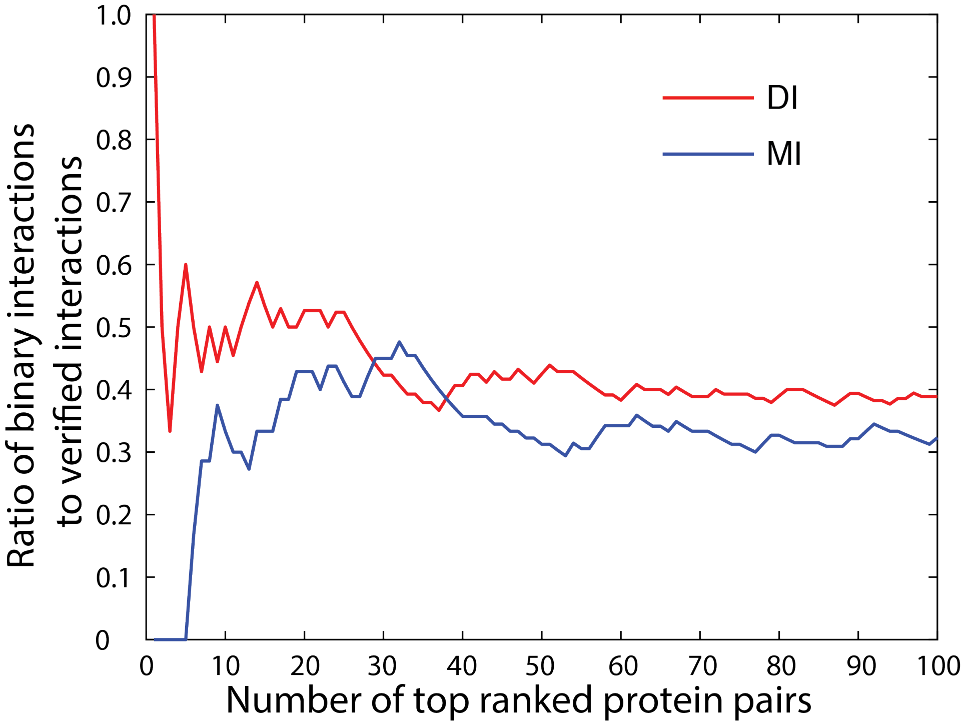

Figure S2. Proportion of binary interactions in the set of positive interactions as a function of top ranked pairs. The ratios of binary interactions to positively predicted interactions verified by literature and STRING database (y-axis) are plotted as total protein pairs ranked by DI values (red) or MI (blue) values.

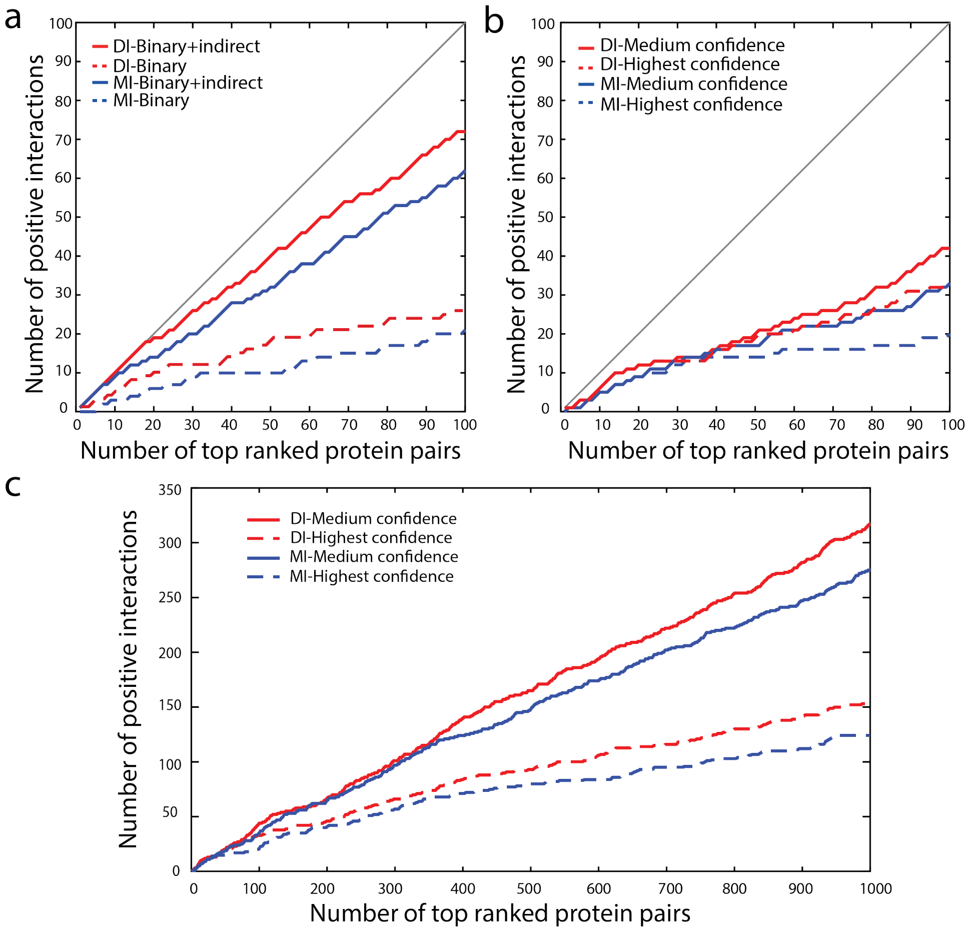

Figure S3. Comparison of top 100 protein-protein pairs predicted from direct information and mutual information by using literature verification and STRING database. (a) The protein-protein pair interaction is verified by a curated search in publications. Solid line plots indicate number of positive protein-protein pairs against total number of pairs (x-axis) ranked by DI values (red) or MI (blue) values. Dash line plots indicate coupling pairs identified as *binary interactions* classified by manual verification. **(b) and (c)** The protein-protein pair interaction is verified by searching interactions at STRING. Number of protein-protein pairs (y-axis) recorded in STRING database against total number of pairs (x-axis) ranked by DI (red) values or MI (blue) values. Highest confidence: combined score ≥ 0.9 in STRING. Medium confidence: combined score ≥ 0.4.

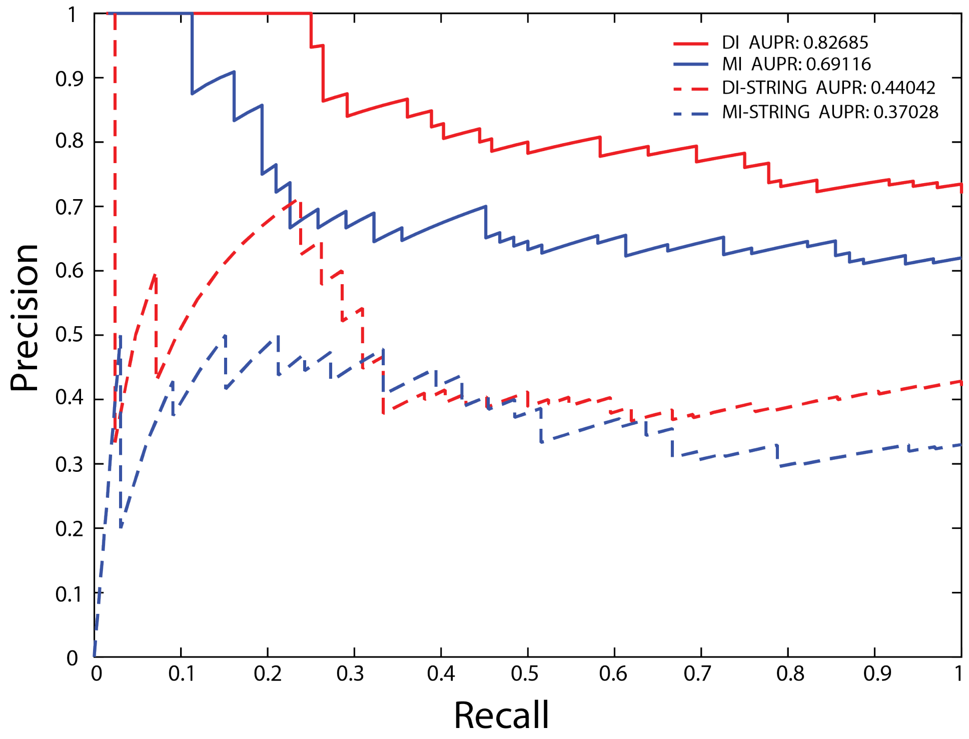

Figure S4. Precision-recall (PR) curves for direct information and mutual information predictions for protein-protein pairs. The total protein pairs for computing precision and recall values are the top 100 pairs. PR curves are constructed by cumulatively increasing the threshold by selecting a ranking number from 1 to 100 and then computing the precision and recall values respectively. Solid plots are computed by using protein pairs validated using literature search as the true interaction set, while dashed are computed by using protein pairs validated against STRING database as the true interaction set. Performance of DI and MI under two different validation strategies is quantified with the area under the precision-recall curve (AUPR).

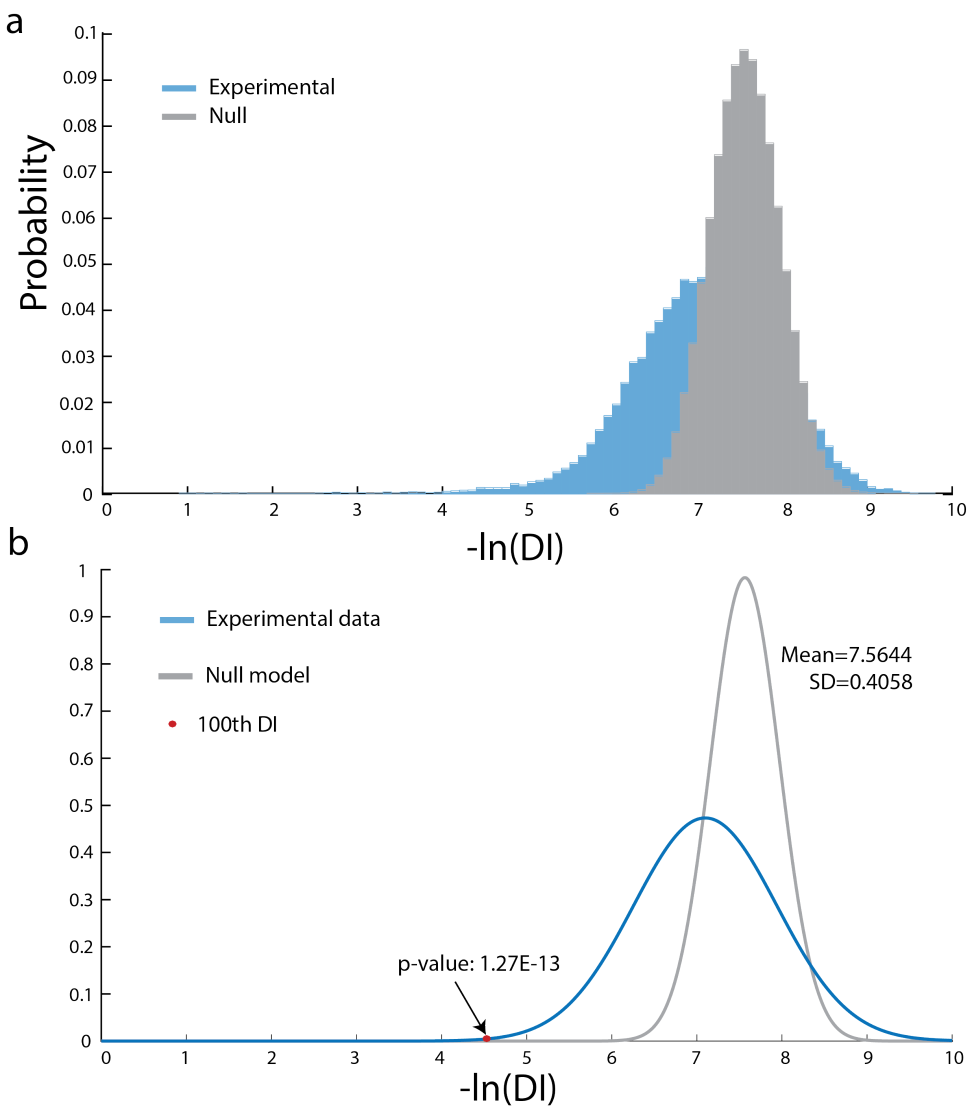

Figure S5. Comparison of direct information distributions computed from a protein expression dataset and a null model. (a) Histogram of DI values computed by using experimental dataset, which is the discretized protein expression levels, and of natural logarithm of DI values computed by using a null model, constructed by randomly shuffling experimental dataset for 100 iterations. Values on the x-axis are computed by taking a negative natural logarithm of DI values. (b) Fitted normal distribution for natural logarithm of null DI values. Means and standard deviations for the null model distributions are shown in the plot. The distribution of experimental DI values is also plotted to show the disassociation from null model. The location of DI value for 100th ranked protein pair is shown as a red dot, with a p-value of 1.27E-13, left tail z-test.

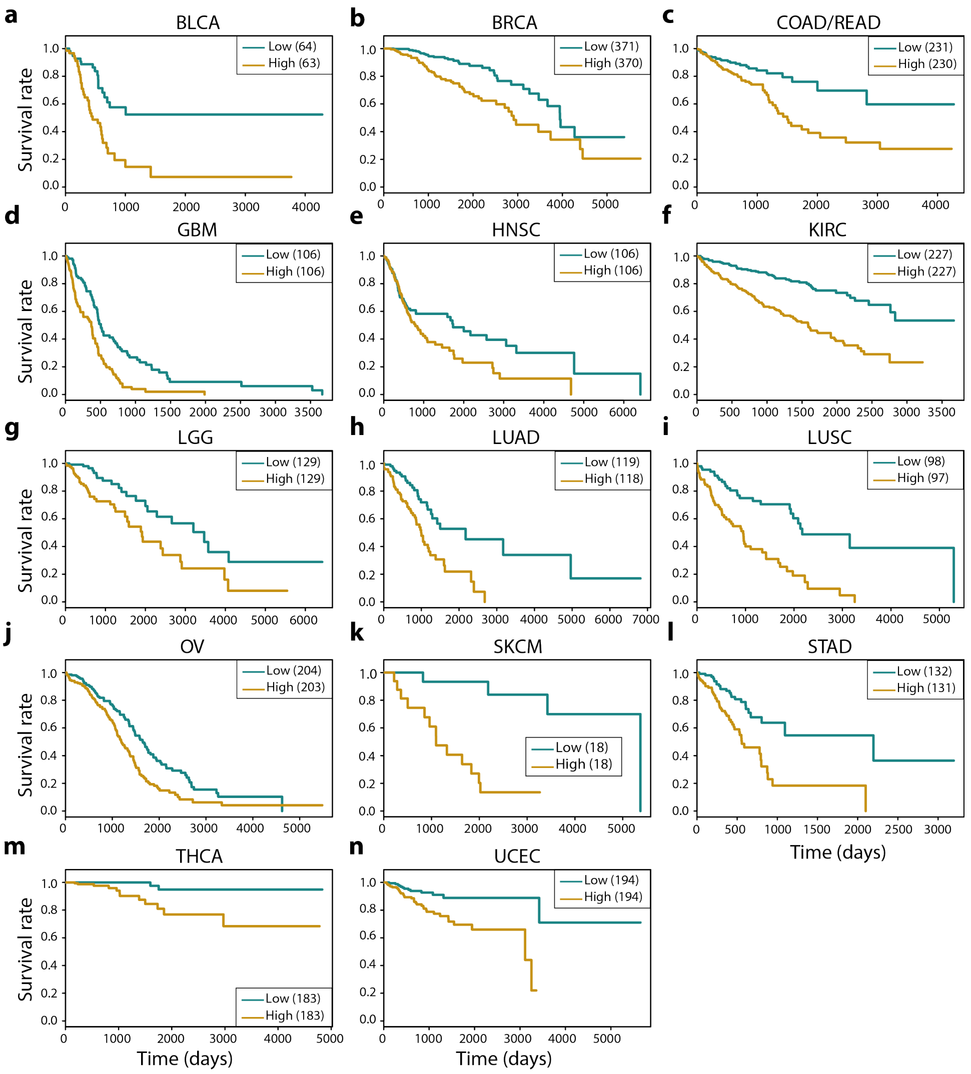

Figure S6. Survival analysis Kaplan-Meier curves using 20 proteins as features on 15 types of cancers. (a) BLCA (*P* =6.04E-05, log-rank test; CI=0.710; HR=3.12). (b) BRCA (*P* = 8.66E-06, log-rank test; CI=0.705; HR=2.51). (c) COAD/READ (*P* =3.58E-04, log-rank test; CI=0.614; HR=2.19). (d) GBM (*P* =1.58E-06, log-rank test; CI=0.630; HR=2.18). (e) HNSC (*P* =2.14E-02, log-rank test; CI=0.574; HR=1.53). (f) KIRC (*P* =1.05E-10, log-rank test; CI=0.707; HR=2.92). (g) LGG (*P* =6.97E-04, log-rank test; CI=0.762; HR=2.45). (h) LUAD (*P* =4.41E-05, log-rank test; CI=0.661; HR=2.62). (i) LUSC (*P* =5.18E-07, log-rank test; CI=0.689; HR=3.19). (j) OV (*P* =1.17E-04, log-rank test; CI=0.607; HR=1.71). (k) SKCM (*P* =4.72E-05, log-rank test; CI=0.872; HR=12.28). (l) STAD (*P* =1.86E-05, log-rank test; CI=0.668; HR=2.86). (m) THCA (*P* =1.21E-03, log-rank test; CI=0.828; HR=8.04). (n) UCEC (*P* =2.98E-04, log-rank test; CI=0.676; HR=3.15). Blue line indicates patients with low risk while yellow line represents high-risk patients. The numbers of patients are shown in the parentheses. HR: mean hazard ratio. CI: concordance index. In most cancers, the low risk group and high risk group are separated well, especially in SKCM, BLCA.

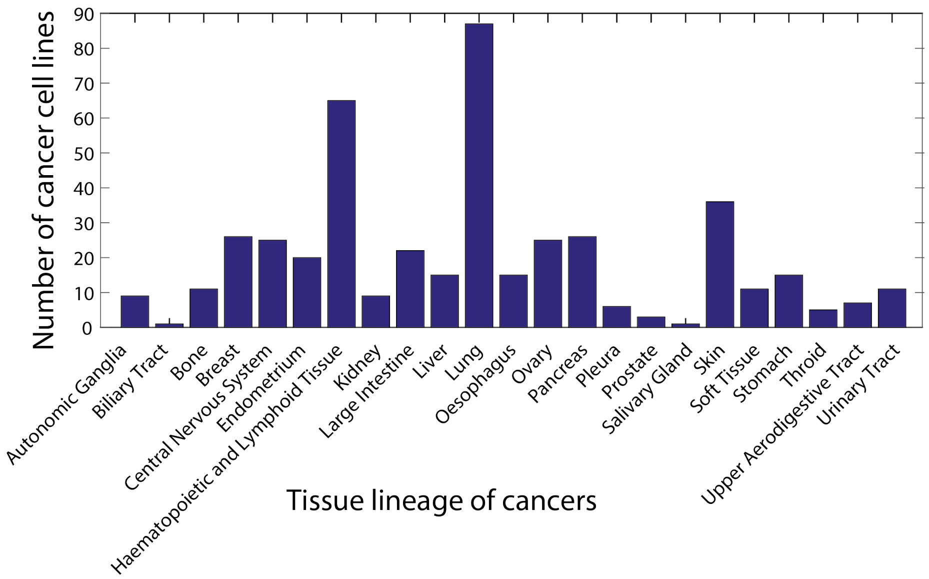

Figure S7. Tissue lineage distribution of cancer cell lines in CCLE dataset. The 451 cancer cell lines in the pharmacogenomics data cover 23 types of tissue lineages (x-axis). Y-axis shows the number of samples for each type of cancer cell line.

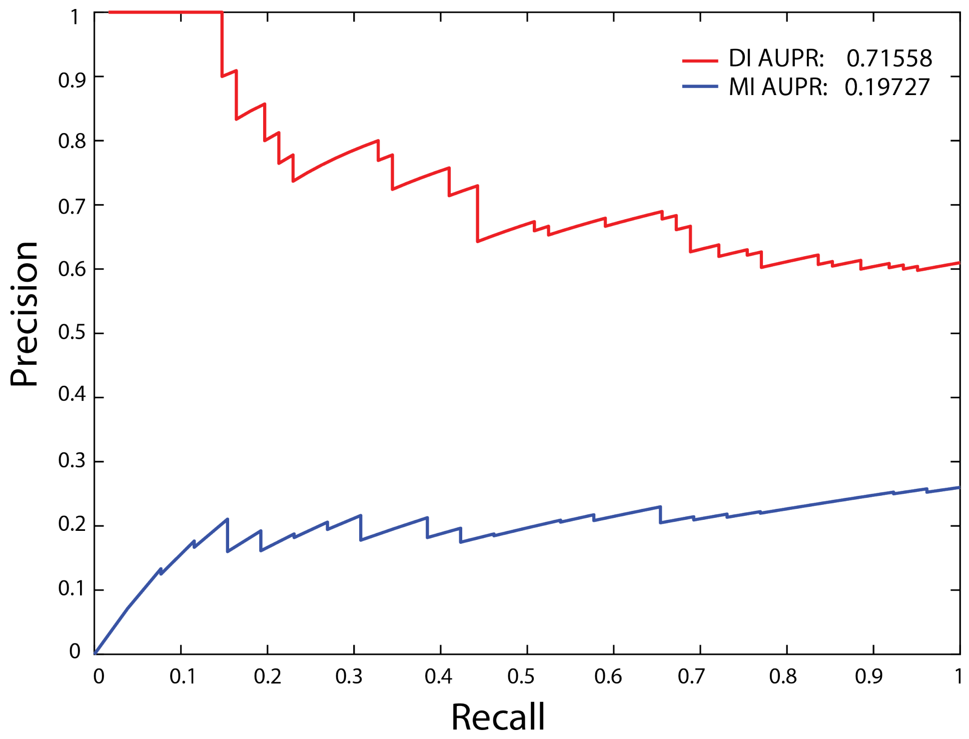

Figure S8. Precision-recall (PR) curves for direct information and mutual information predictions for gene mutation-drug response dataset. PR curves are constructed by cumulatively increasing the threshold by selecting a ranking number from 1 to 100 and then computing the precision and recall values respectively. Performance of DI and MI method is quantified with the area under the precision-recall curve (AUPR).

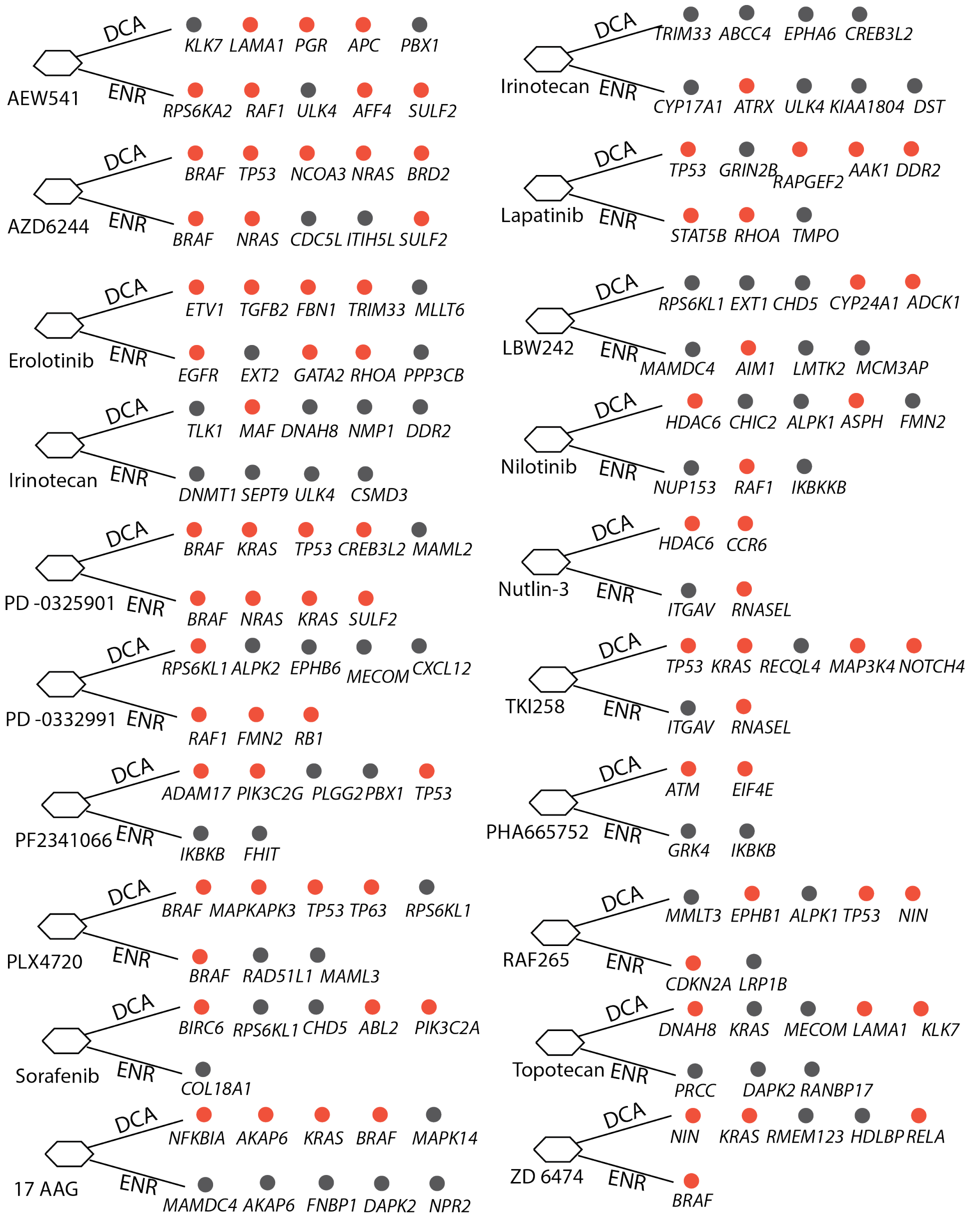

Figure S9. Comparison between DCA and elastic net regression method. A maximum of 5 connected genes for drugs uncovered both by DCA (ranked among top 1%) and elastic net regression (ENR) are shown next to each drug with orange color representing positive prediction and grey color denoting negative prediction. The average positive prediction rates for DCA and ENR are 63% and 41%, respectively.

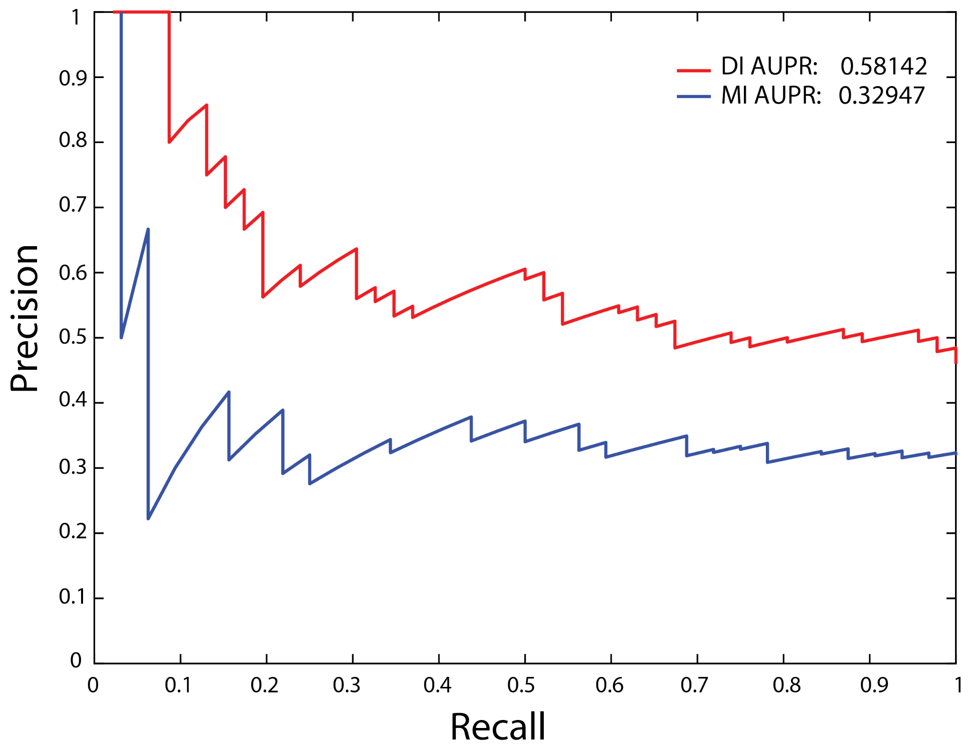

Figure S10. Precision-recall (PR) curves for direct information and mutual information predictions for gene expression-drug response dataset. PR curves are constructed by cumulatively increasing the threshold by selecting a ranking number from 1 to 100 and then computing the precision and recall values respectively. Performance of DI and MI method is quantified with the area under the precision-recall curve (AUPR).

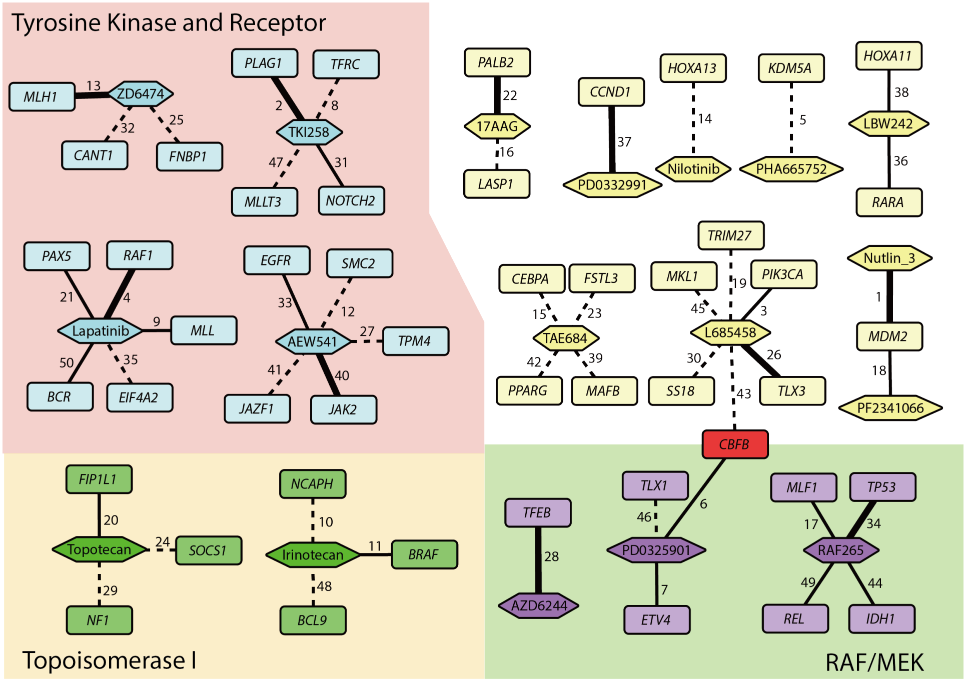

Figure S11. Top 50 drug-gene expression relationships inferred with direct information. Clusters are annotated with different colors based on the pathways to which the targets of drugs belong. Rectangles represent drugs and ellipses are genes. Red nodes indicate gene hubs connecting to several drugs. Solid lines indicate that the gene product is known to relate with the target of the drug it connects, defined as *known interactions*, with thick solid edges indicating *strong interactions* between drug and gene. The thin solid lines indicate a weaker relationship between drug and gene. Dashed edges show *predicted interactions* between drug and gene that have not been described in the literature. The edge coefficients represent the rank using direct information as a metric.
